# Supplementary material for: Learning curve and surgical outcome of robotic assisted colorectal surgery with ERAS program
Source: Sci Rep. 2022 Nov 29;12:20566. doi: 10.1038/s41598-022-24665-w (PMC9709162; doi:10.1038/s41598-022-24665-w)
Supplement: Supplementary file 1 — Supplementary Information 1. [file 41598_2022_24665_MOESM1_ESM.zip › LSC Raw data-Ñ[▒K/099/P_ñw╝╨░O▒Kñσ.pdf]

[redacted]

Pathologic diagnosis:
1. Sigmoid colon, laparoscopic anterior resection --- Status post biopsy with no residual tumor seen. The bilateral cut ends and mesocolic lymph nodes (0/13) are free of tumor.
2. Colon, cut end, distal, resection --- Unremarkable.

Prognostic and predictive factor:
1. Tumor size: Cannot be assessed.
2. Depth of invasion: Cannot be assessed.
3. Histologic grade: Not applicable.
4. Angiolymphatic invasion: Not identified.
5. Perineural invasion: Not identified.
6. Tumor deposits (discontinuous extramural extension): Not identified.
7. Macroscopic tumor perforation: Not identified.
8. Surgical Margins:
a) Margin near the dyed area: Uninvolved by invasive carcinoma, distance: 3.7 cm.
b) Margin far from the dyed area: Uninvolved by invasive carcinoma, distance : 10.5 cm.
c) Radial or Mesenteric Margin: Uninvolved by invasive carcinoma, distance : 10 cm.
9. Lymph node status: Uninvolved (0/13).
10. Extranodal involvement: Not applicable.
11. Tumor regression grading S/P CCRT: No known presurgical therapy.
12. Type of polyp in which invasive carcinoma arose: Not applicable.
13. Additional pathologic findings: Not identified.
14. Pathological TNM Stage: ypTxN0(According to the eighth edition, American Joint Committee on Cancer Staging Guidelines for Tumors).
15. TNM descriptors: y (Post-treatment).

Gross description:
The specimen consists of 1) a segment of colon, 19 cm in length and 3.8/4.2 cm in bilateral cut end circumferences, and mesocolic soft tissue. Area of blue dye is noted, 5x2.5 cm, with a small pin, is noted, 3.7 cm to the nearest cut end. 2) a piece of tan gray soft tissue, 2x1.8x0.6 cm, labeled as "distal cut end". All for section: A) specimen 2; Representative part for section: B) cut end near the lesion C) cut end far from the lesion D-L) dye lesion M) radial cut end N-P) LN.
#T-59300_2 #M-09183_2 0 1105 00SP00
#T-C4560_2 #M-09183_2 0 1105 000000

已電子簽章

敬告：本單僅供醫師參考使用，不具任何申請效力！！
請病患妥為保管，若不慎遺失需自負個人資料外洩責任！
